# Macrophage-tumor cell interactions regulate the function of nitric oxide

**DOI:** 10.3389/fphys.2013.00144

**Published:** 2013-06-18

**Authors:** Michal A. Rahat, Bernhard Hemmerlein

**Affiliations:** ^1^Department of Immunology, Immunology Research Unit, Carmel Medical Center and the Ruth and Bruce Rappaport Faculty of MedicineTechnion, Haifa, Israel; ^2^Department of Pathology, Georg-August University Hospital, Göttingen, and the HELIOS-KlinikumKrefeld, Germany

**Keywords:** inducible nitric oxide synthase (iNOS), tumor cells, macrophage activation, apoptosis, angiogenesis, miR-146a, macrophage-induced death, macrophage therapy

## Abstract

Tumor cell-macrophage interactions change as the tumor progresses, and the generation of nitric oxide (NO) by the inducible nitric oxide synthase (iNOS) plays a major role in this interplay. In early stages, macrophages employ their killing mechanisms, particularly the generation of high concentrations of NO and its derivative reactive nitrogen species (RNS) to initiate tumor cell apoptosis and destroy emerging transformed cells. If the tumor escapes the immune system and grows, macrophages that infiltrate it are reprogramed *in situ* by the tumor microenvironment. Low oxygen tensions (hypoxia) and immunosuppressive cytokines inhibit iNOS activity and lead to production of low amounts of NO/RNS, which are pro-angiogenic and support tumor growth and metastasis by inducing growth factors (e.g., VEGF) and matrix metalloproteinases (MMPs). We review here the different roles of NO/RNS in tumor progression and inhibition, and the mechanisms that regulate iNOS expression and NO production, highlighting the role of different subtypes of macrophages and the microenvironment. We finally claim that some tumor cells may become resistant to macrophage-induced death by increasing their expression of microRNA-146a (miR-146a), which leads to inhibition of iNOS translation. This implies that some cooperation between tumor cells and macrophages is required to induce tumor cell death, and that tumor cells may control their fate. Thus, in order to induce susceptibility of tumors cells to macrophage-induced death, we suggest a new therapeutic approach that couples manipulation of miR-146a levels in tumors with macrophage therapy, which relies on *ex vivo* stimulation of macrophages and their re-introduction to tumors.

## Immunoediting: interplay between macrophages and tumor cells

Tumors arise when tissue cells accumulate genetic alterations/mutations that disrupt the tightly controlled cell growth and division systems (Hanahan and Weinberg, 2011), leading to uncontrolled proliferation of these cells and increased tumor mass. After overcoming intrinsic tumor-suppressor mechanisms (Vesely et al., 2011), the cells have to evade the immune system. In fact, most of the time the immune system succeeds in eliminating those aberrant cells, in a process once known as immunosurveilence (Dunn et al., 2004; Vesely et al., 2011). To better describe the complex interactions between tumor cells and the immune system, the term “immnoediting” has been coined (Dunn et al., 2004; Bui and Schreiber, 2007; Reiman et al., 2007; Schreiber et al., 2011) and consists of three stages; In the first stage (the elimination stage, previously known as immunosurveilence) immune cells destroy emerging transformed cells and prevent their development into a tumor. If this process is unsuccessful, there is a transition period to the second phase (equilibrium), where the immune system is able to contain but not eliminate the tumor. During equilibrium, the tumor cells are under constant immune pressure that eliminates many of the original variants but additional mutations may allow for new variants to be generated. Eventually, some variants may escape from the immune pressure triggering the third phase (escape), and becoming free to grow in an immunologically unrestricted manner. This sequence of events means that there is a constant and dynamic interplay between the tumor cells and the stroma immune cells, which continuously changes according to the shift in conditions. Among the immune cells, macrophages are the most prominent, as they infiltrate deep into the low oxygen tension (hypoxic) regions of the tumor and accumulate there, so that in some cases they can make up as much as 50% of the tumor mass (Murdoch et al., 2004; Mantovani et al., 2008).

Generally, macrophages are cells known to infiltrate tissues in order to combat and eradicate invading pathogens and tumor cells. Actually, they have additional tasks, including patrolling their surroundings and maintaining homeostasis, orchestrating tissue healing and repair and resolving inflammation. It is obvious that such opposing functions cannot be simultaneously performed by the same macrophage, and therefore, it was suggested that macrophages can be differentially activated depending on the signals they receive from their immediate microenvironment. Thus, macrophages display enormous plasticity (Stout and Suttles, 2004; Stout et al., 2009), and can shift from one activation mode to another, unlike lymphocytes that remain committed to only one kind of activation. This concept has been extensively reviewed before (Mosser and Edwards, 2008; Murdoch et al., 2008; Martinez et al., 2009; Gordon and Martinez, 2010; Qian and Pollard, 2010) and will be only briefly mentioned here.

In recent years many studies have shown that during the escape phase macrophages become supportive and even critical to tumor progression, growth and metastasis, as they produce growth factors, cytokines and chemokines which are necessary for these processes. In order to escape immune killing, tumor cells activate several mechanisms to control the immune response, which include acquiring defects in the antigen processing and presentation pathways to facilitate evasion from adaptive immune recognition (Rabinovich et al., 2007), secretion of immunosuppressive mediators (e.g., TGFβ, IL-10, IL-13, PGE_2_), and recruitment of regulatory immune cells (Bui and Schreiber, 2007; Rabinovich et al., 2007).

In this review we focus on the interplay between tumor cells and macrophages during different stages of tumor development, as manifested by the complex roles of a single molecule—inducible nitric oxide synthase (iNOS) and its product nitric oxide (NO). We review the conditions that regulate its expression and activity in different cell types and in changing microenvironments, and explore the significance of these differences. Finally, we describe possible future approaches that explore whether these interactions can be modulated in order to manipulate expression of iNOS or its activity, and to effectively enhance tumor eradication.

## NO production in different types of macrophage activation

Generally, three types of macrophage activation can be described. Classically activated or M1 macrophages are activated by ligands of toll-like receptors (TLRs) and pro-inflammatory cytokines (e.g., interferon γ—IFNγ, tumor necrosis factor α—TNFα, interleukin 1β—IL-1β), they activate the Th1 immune response and secrete high amounts of pro-inflammatory mediators that kill the invading pathogens or tumor cells, such as the cytotoxic TNFα and NO. In fact, the high expression of the iNOS that produces NO is the hallmark of these macrophages (Mosser, 2003; Mosser and Zhang, 2008). Alternatively or M2 activated macrophages are activated by and secrete anti-inflammatory mediators (e.g., IL-10, IL-13, tumor growth factor β—TGFβ, and prostaglandin E_2_—PGE_2_), which together generate a microenvironment that suppresses the activity of M1 macrophages and Th1 lymphocytes. M2 macrophages are involved mainly in homeostasis, tissue remodeling and wound healing, as they remove cellular debris, support phagocytosis (by expressing scavenger receptors. the mannose receptor CD206), and deposit extracellular matrix (ECM) proteins (e.g., fibronectin). M2 macrophages express high levels of arginase-I, which produces ornithine, the precursor of collagen. Arginase-I also competes with iNOS for their common substrate L-arginine, and prevents NO production (Martinez et al., 2009; Gordon and Martinez, 2010). Regulatory macrophages, a third type of activation, can be activated by TLR and immune complexes, by anti-inflammatory cytokines or mediators (e.g., adenosine), or by phagocytosis of apoptotic cells (Mosser, 2003; Mosser and Edwards, 2008). While several subtypes of regulatory activations have been identified, all types inhibit pro-inflammatory reactions, partly by secreting anti-inflammatory cytokines (e.g., IL-10, TGFβ). As macrophages exhibit great plasticity, they may exhibit additional types of activation within the range defined by these three main types to yield many different sub-populations with different roles and functions (Mosser and Edwards, 2008).

Three main macrophage subsets have been identified within the tumor mass and can be localized in different niches of the tumor (Lewis and Pollard, 2006). Tumor-associated macrophages (TAMs) support tumor progression and metastasis, as they secrete pro-angiogenic growth factors (e.g., vascular endothelial cell growth factor—VEGF), and matrix metalloproteinases (MMPs). TAMs infiltrate deep into the tumor and are found in perinecrotic and hypoxic areas. In addition to the secretion of many pro-angiogenic factors (e.g., FGF2, IL-8, PDGF, VEGF, MMP-7, and MMP-12), TAMs also use several mechanisms to render M1 macrophages as well as CD4^+^ and CD8^+^ T cells non-responsive to tumor-specific antigens, including secretion of immunosuppressive mediators (e.g., IL-10) and depletion of L-arginine by the activity of arginase-I (Coffelt et al., 2009). Moreover, TAMs are necessary for metastasis, and their ability to secrete EGF together with the ability of tumor cells to secrete M-CSF/CSF-1 stimulate mutual migration in both cell types (Wyckoff et al., 2004; Condeelis and Pollard, 2006; Coffelt et al., 2009; Hernandez et al., 2009).

Tie2-expressing monocytes (TEMs), that unlike TAMs reside very close to blood vessels (Venneri et al., 2007), are similar to TAMs in their support for tumor progression and metastasis via pro-angiogenic growth factors such as VEGF and MMPs. In fact, TEMs are essential for tumor progression, as their depletion markedly inhibits tumor angiogenesis (De Palma et al., 2005; Venneri et al., 2007). In addition, TEMs are potent immunosuppressive cells, as they can secrete high levels of IL-10, suppress T cell proliferation and promote the expansion of regulatory T cells (Treg) (Coffelt et al., 2011).

In tumor-bearing mice and humans, expanded populations of myeloid-derived suppressor cells (MDSCs) are found within the tumors, spleen and bone marrow in proportion to the tumor size. MDSCs are a mixture of immature granulocytic and monocytic cells, and monocytic MDSCs belong to the regulatory macrophages. MDSCs are triggered by a combination of IFNγ and IL-13, and secrete IFNγ, IL-13, IL-10, and TGFβ, which help them suppress Th1 cell-mediated immune response, induce regulatory T cells and inhibit M1 macrophages (Bronte, 2009; Gabrilovich and Nagaraj, 2009; Ostrand-Rosenberg and Sinha, 2009). Production of NO and peroxynitrite help MDSCs to nitrate the TCR and CD8 molecules on cytotoxic T cells, inhibiting the ability of the latter to bind to MHC class I molecules and rendering them non-responsive (Nagaraj et al., 2007; Nagaraj and Gabrilovich, 2008).

TAMs, TEMs, and MDSCs express similar activation markers, which place them between regulatory macrophages and M2 macrophages (De Palma et al., 2007; Mosser and Edwards, 2008; Murdoch et al., 2008). More accurately, these macrophages exhibit a mixed expression profile, of both M1 and M2 markers. In this respect, TEMs are considered more M2-skewed than TAMs, as they express more arginase-I but less iNOS (Pucci et al., 2009), whereas MDSCs express both arginase-I and iNOS (Corzo et al., 2010). It is possible that these subsets represent different linages that develop separately (Pucci et al., 2009), or they may gradually progress from one to the other, as they migrate from the blood vessels into the perinecrotic areas and continue to be polarized or reprogrammed by the local tumoral microenvironment that consists of a gradient of cytokines and hypoxia, and by the interactions with the tumor cells.

## The multiple biological roles of NO in tumors

NO is a small, short-lived, lipophilic gas molecule, which can easily cross membranes, and rapidly reacts with oxygen or superoxide to generate the derivatives that exert its biological activity. NO has been shown to both promote and inhibit tumor growth and metastasis. Although first recognized as a cytotoxic molecule that serves as a major killing mechanism of macrophages during pathogen infection or tumor cell killing, it also functions as a regulator of wound healing, tissue repair and suppression of the immune response-properties required to promote tumor growth. In fact, the different levels of iNOS expression in TAMs, TEMs, and MDSCs suggest multiple roles.

NO is produced by three isoforms of nitric oxide synthase (NOS) that convert L-arginine to L-citrulline. The endothelial (eNOS/NOS3) and neuronal (nNOS/NOS1) isoforms produce low amounts of NO (in the pM-nM range), and produce only a small fraction of the total NO in tumors. The bulk of NO in tumors is produced by the high output inducible isoform (iNOS/NOS2), which is strongly induced in macrophages and in tumor cells, and produces high concentrations of NO (in the μM range) (Xie and Nathan, 1994). It is important to note that unlike other inflammatory mediators that need to be enzymatically modulated or degraded, NO can chemically and directly react with other molecules (e.g., oxygen, superoxide) to produce multiple derivatives. Some of these derivatives are relatively stable (e.g., nitrites, hydroxylamine), and some are reactive nitrogen species (RNS) (e.g., peroxynitrite, nitrogen dioxide, nitroxyl) that are also biologically active (Donzelli et al., 2006). More details on the complex NO chemistry can be found elsewhere (Lechner et al., 2005; Wink et al., 2011). Since it is very difficult to separate between the effects of NO and its active derivatives, we will refer to them collectively as NO/RNS.

**Figure 1:**
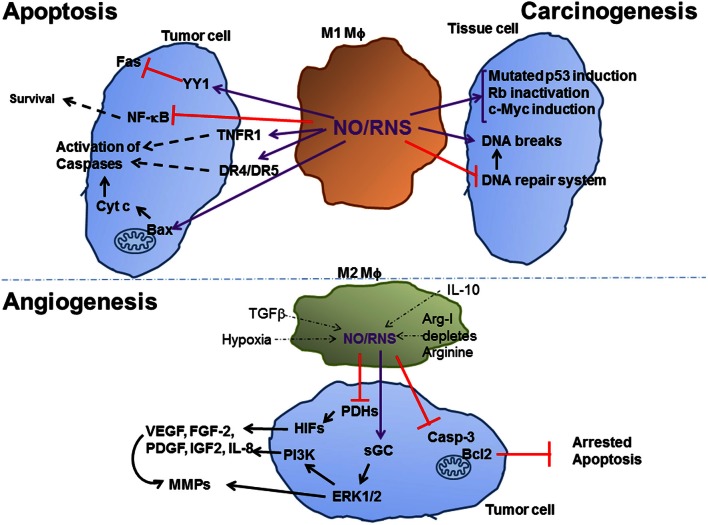
**Effect of Macrophage NO on tumor carcinogenesis, apoptosis and angiogenesis**. M1-activated macrophages that produce high amounts of NO/RNS help carcinogenesis by increasing DNA breaks and mutations, arresting the DNA repair system and inducing oncogenes or inactivating tumor suppressor genes. High levels of NO/RNS may also drive apoptosis of established tumor cells, by modifying death receptors an increasing their expression, by inhibiting NF-kB and by enhancing cytochrome c release from the mitochondria. Low levels of NO/RNS produced in different subsets of M2-activated macrophages, and are reduced due to the effects of inhibitory microenvironmental factors, such as hypoxia. Hypoxia and NO/RNS stabilize the HIF family of transcription factors, and activate the MAPK ERK1/2 and the PI3K pathways, thus inducing the expression of pro-angiogenic factors, as described in the text. Purple arrows indicate post-translation modification (e.g., S-nitrosylation) of proteins, black arrows indicate activation of a protein/process, dashed arrows indicate a multi-step process, thin dashed arrows indicate the inhibitory effects of microenvironmental factors on NO/RNS production.

Depending on their concentrations, NO/RNS react with DNA, proteins and lipids and can act either as a signaling molecule that initiates signaling pathways or as a molecule that causes damage. Depending on the balance with other ROS, especially superoxide, NO/RNS may deaminate the DNA bases guanine, cytosine and adenine, causing DNA breaks and mutations, or it can affect proteins in one of four ways: (1) oxidation of metal prosthetic groups (heme or non-heme); (2) nitration, the covalent attachment of a nitro group (Tyr–NO_2_) to tyrosine and tryptophan residues; (3) S-nitrosylation of thiol and amine groups, which covalently attaches NO to form a weak and reversible S-nitrosothiol (S-NO) bond; (4) oxidation of thiol groups in cysteine and methionine residues, that yield intramolecular disulfide bonds (-S-S-), cysteine sulfenic acid (R-S-OH), sulfinic acid (R-SO_2_H) or sulfonic acids (R-SO_3_H) (Lala and Chakraborty, 2001; Leon et al., 2008). These post-translational modifications can potentially activate or inhibit target proteins, with different biological consequences. The final biological outcome depends on the NO concentrations produced, on the cellular redox state and bioavailability of other ROS, on the cellular location of production, on the distance of the impacted proteins from the generated NO, and on the cell type (Leon et al., 2008). Research conducted with NO donors revealed threshold concentrations of NO/RNS that are needed to activate specific pathways. For example, 50 nM of NO were sufficient to phosphorylate ERK, 100 nM stabilized HIF-1α and activated the Akt pathway, more than 300 nM were required to cause DNA damage and induce p53 and 1 μM was considered nitrosative stress (Wink et al., 2011). In tumors, NO was described to have both pro- and anti-tumoral effects, depending first and foremost on its concentrations (summarized schematically in Figure 1). As these aspects have been extensively reviewed elsewhere (Lala and Chakraborty, 2001; Lechner et al., 2005; Lancaster and Xie, 2006; Weigert and Brune, 2008), we will only briefly mention these here.

### Tumor inhibiting activities

In general, high concentrations of NO/RNS can arrest cell cycle (cytostatic effect) or induce death, whereas low concentrations may protect cells from death. In fact, generation of high levels of NO/RNS is a very effective tool to induce death, and macrophages use it as a major weapon in their arsenal against invading pathogens and tumor cells (Weigert and Brune, 2008). High levels of NO/RNS post-transnationally modify death-related target proteins, and could mediate inhibition of cellular respiration in target cells, leading to their cell cycle arrest.

Modification of death receptors of the TNFα superfamily (e.g., Fas, TRAIL, and TNFRI, DR4, and DR5), or of mitochondrial targets that affect the mitochondrial respiratory chain and its outer membrane permeability leading to the release of cytochrome c and initiation of apoptosis, are the two main pathways leading to cell death [extensively reviewed in Lechner et al. (2005), Jeannin et al. (2008), Leon et al. (2008)]. Thus, S-nitrosylation of the YY1 transcription factor alleviates its suppression on the Fas promoter, resulting in increased apoptosis, and NO-donors enhance apoptosis by increasing the expression of TNFα receptors. NO/RNS can bind to the heme-copper center of the reduced form of cytochrome c oxidase, compete for the binding of oxygen, and cause inhibition of the mitochondrial respiratory chain and finally increased mitochondrial membrane permeability and release of cytochrome c (Brune, 2003; Jeannin et al., 2008).

Additional mechanisms may support initiation of apoptosis. For example, NO/RNS may enhance phosphorylation of serine residue 15 of the wild type p53, causing its activation and increased nuclear retention, thereby initiating apoptosis (Brune, 2003; Jeannin et al., 2008), as well as transiently and reversibly down-regulating mdm2, thus contributing to p53 activation (Brune, 2003). NO/RNS in amounts that favor generation of peroxynitrite and DNA damage, lead to accumulation of nitrated p53, improve its DNA binding and cause apoptosis (Leon et al., 2008). Another example is the S-nitrosylation of the p50 subunit of NF-κB on cysteine residue 62 that inhibits its DNA binding activity and reduces NF-κB activity, which is generally considered an anti-apoptotic factor.

### Tumor promoting activities

When discussing the tumor-promoting activities of NO/RNS, it is necessary to distinguish between carcinogenic activities that promote tumor generation, and activities that support a pre-exiting tumor at the stage of escape from the immune system.

#### Carcinogenesis

Chronic inflammation is linked to tumors and is recognized as a predisposing factor for malignant transformation of tissue cells (Kundu and Surh, 2008). In particular, the high amounts of ROS and RNS that are generated by recruited macrophages and neutrophils, as part of their killing mechanisms, play an important role. Since NO/RNS are lipophilic and can easily cross membranes, tissue cell DNA is exposed to the high concentrations, which may oxidize and/or deaminate the DNA bases, especially during transcription or replication where single strand DNA is more prevalently found. This may result in DNA breaks, DNA base modifications or DNA cross-links, which cause mutations, and may activate oncogenes or deactivate tumor suppressor genes (Lechner et al., 2005; Kundu and Surh, 2008). In addition, NO/RNS-driven protein modifications such as S-nitrosylation or nitration may inhibit proteins belonging to the DNA repair systems and hamper attempts to correct mutations. Thus, NO/RNS drive genomic instability.

In addition, there are many isolated examples for NO-driven protein modifications that further explain the carcinogenic effects of NO/RNS. For example, a negative feedback loop exists between iNOS and p53. NO activates wild-type p53, which is itself a negative regulator of iNOS that binds to its promoter and inhibits iNOS transcription. However, in tumors, p53 is often mutated and cannot inhibit iNOS expression (Lechner et al., 2005). Thus, in a model of chronic inflammation in p53 knockout mice, increased NO production accelerated spontaneous tumor development, compared to the control mice (Hussain et al., 2008). NO-driven hyperphosphorylation of retinoblastoma (Rb) inactivated this tumor suppressor gene in a model of mouse colitis, (Ying et al., 2007). High amounts of NO induced the expression of c-Myc in a breast cancer cell line (Glynn et al., 2010), and activated EGFR and Src in estrogen receptor negative breast cancer patients through S-nitrosylation (Switzer et al., 2012). All of these examples provide a link between chronic inflammation, sustained production of NO/RNS and carcinogenesis.

#### In the escape phase

When tumor cells are already at the escape phase, they employ many pathways to maintain low levels of NO/RNS, as low amounts of NO/RNS are actually beneficial to the tumor cells. Anti-inflammatory mediators in the tumor microenvironment (e.g., TGFβ) reduce transcription of iNOS mRNA and effectively lower production of NO. Arginase-I that is highly expressed by the TAMs and MDSCs depletes L-arginine and leaves insufficient amounts of this common substrate for iNOS activity (Heller, 2008; Wink et al., 2011). In addition, if NO is secreted from TAMs, it is likely to be captured by red blood cells, where it S-nitrosylates their glutathione and hemoglobin, resulting in additional decrease in NO/RNS concentrations (Heller, 2008). Moreover, although hypoxia increases the expression of the iNOS mRNA and protein through the transcription factors HIF-1α and NF-κB, the hypoxic microenvironment actually inactivates the enzyme activity (Melillo et al., 1996), either because of the lack in the enzyme substrate or due to disruption of its protein–protein interactions with α-actinin-4 (Daniliuc et al., 2003). Collectively, these mechanisms ensure that only low amounts of NO/RNS are generated within the tumoral microenvironment.

Low amounts of NO/RNS are anti-apoptotic and beneficial for tumor cells. S-nitrosylation of caspases, especially caspase-3, inhibits the enzymes, and blocks apoptosis (Leon et al., 2008). S-nitrosylation of the FLIP adaptor protein prevents its Fas-induced ubiquitination and degradation and enables it to exert its anti-apoptotic activity (Iyer et al., 2008). Similarly, S-nitrosylation of Bcl2 also protected this protein from ubiquitination and degradation (Iyer et al., 2008). Thus, low amounts of NO/RNS which activate S-nitrosylation of proteins may be a general mechanism to prevent degradation of anti-apoptotic proteins and protect cells from death.

***The role of NO in Angiogenesis.*** Angiogenesis, the process in which vascular endothelial cells proliferate and reorganize to form new vessels sprouting from pre-existing blood vessels, is essential for the growth of most primary tumors and their subsequent metastasis. Hypoxic core regions in tumors, which lack oxygen and nutrients, initiate the process of angiogenesis to generate growth of new blood vessels into the tumor. Many pro-angiogenic factors, including the most potent regulator and pivotal mediator VEGF, as well as FGF-2, PDGF, IGF2, TGFβ, and IL-8, are all induced by hypoxia inducible factor 1 or 2, which are transcription factors that bind to the hypoxia response element (HRE) located in the promoters of these genes (Black et al., 2008; Wink et al., 2011; Chowdhury et al., 2012).

Both hypoxia (<5% O_2_) and NO/RNS can stabilize HIF-1α and HIF2α Both HIF-α subunits are constitutively transcribed and translated, but immediately directed for degradation in normoxia, through their hydroxylation of proline residues by the prolyl hydroxylases (PHDs) that rely on oxygen as their substrate. This hydroxylation recruits the von Hippel Lindau (VHL) protein, which has an E3 ubiquitin ligase activity that marks HIF-α subunits for degradation in the proteasome. Hypoxia inactivates PHDs due to the limited oxygen substrate, and therefore stabilizes the HIF-α subunits, allowing their heterodimerization with the HIF-1β subunit (Nizet and Johnson, 2009; Walmsley et al., 2009; Rahat et al., 2011). Low levels of NO/RNS can also stabilize HIF proteins by inactivating PHDs through oxidation of their non-heme Fe^+2^-group, thereby causing reduced hydroxylation of HIF-1α and its accumulation even in normoxic regions of the tumor, close to the rims (Kimura et al., 2000, 2001).

Low amounts of NO further promote the induction of these aforementioned pro-angiogenic genes by activating guanylate cyclase and increasing cGMP levels, which help phosphorylate the MAP kinases ERK1/2 and activate PI3K/Akt, that activate additional transcription factors that are needed for the induction of the factors (Dulak and Jozkowicz, 2003; Ridnour et al., 2006). Such pro-angiogenic factors directly affect endothelial cells, as they are growth factors needed for their survival and proliferation, as well as for their spatial reorganization into tube-like formation (Ridnour et al., 2006).

While helping to induce pro-angiogenic factors, NO/RNS suppress the expression of thrombospondin-1 (Tsp1) (Ridnour et al., 2005), which limit angiogenesis by reducing the migration and proliferation of endothelial cells. This cross-talk between NO and Tsp1 is regulated by the concentrations of NO, as low NO levels down-regulate Tsp-1 expression, and increased levels of Tsp-1 inhibit the pro-angiogenic effects of NO (Ridnour et al., 2006).

Low levels of NO/RNS can directly and indirectly via VEGF enhance angiogenesis by activating MMP-1, MMP-9, and MMP-13 (Ridnour et al., 2007; Ziche and Morbidelli, 2009), MMPs are critical for angiogenesis, as they degrade components of the ECM and pave the way for migration of endothelial cells into the tumor, and of tumor cells out of the tumor to the nearest blood vessel. High levels of MMPs, particularly MMP-9, release and activate VEGF that is trapped by the ECM, and allow migration of endothelial cells, as well as leukocytes and metastatic tumor cells. In addition to its direct pro-angiogenic properties, VEGF is also a regulator of MMP-9, thus creating a positive feedback loop whereby MMP-9 and VEGF enhance each other (Hollborn et al., 2007). Low levels of NO/RNS control MMPs by activating JNK and NF-κB (Yang et al., 2011), and simultaneously down-regulate MMP's endogenous inhibitor TIMP-2 (Ziche and Morbidelli, 2009). Reduced levels of TIMP-2 not only allow the activity of MMPs, but are also pro-angiogenic, independently of their effect on MMPs (Lahat et al., 2011).

Thus, low NO/RNS levels enable multiple paths for angiogenesis, and shift the balance between pro- and anti-angiogenic factors to enhance angiogenesis.

***Immune evasion.*** NO/RNS further contribute to the inhibition of anti-tumor immune responses and the ability of tumors to evade the immune system by increasing T cell apoptosis, and by nitrating TCR on CD8+ T cells, thereby inhibiting their ability to kill antigen-specific tumor cells (Ostrand-Rosenberg and Sinha, 2009; Jia et al., 2010). A recent paper now describes an additional role for tumor-produced NO/RNS in attracting MDSCs and inducing their function (Jayaraman et al., 2012), thus enhancing immunosuppression and helping the tumors to evade immune recognition. This study further illustrates the importance of the cell type producing NO/RNS, and its critical role in mediating tumor cell-macrophage interactions.

Thus, tumor cells have a vested interest to lower NO concentrations in the tumor microenvironment. They employ different strategies, including the secretion of immunosuppressive cytokines (e.g., IL-10, TGFβ and PGE_2_), the use of the hypoxic microenvironment that inactivate iNOS activity, and the depletion of L-arginine by arginase-I, to reduce NO production in the infiltrating macrophages. By doing so, tumor cells reprogram macrophages to ensure their pro-angiogenic activation, thus “enslaving” them to the tumor needs.

## Regulation of iNOS expression and NO activity

The regulation of iNOS expression and its activity have been extensively reviewed before (Alderton et al., 2001; Aktan, 2004; Pautz et al., 2010) and we will only briefly describe it here. The main regulatory checkpoint on iNOS expression is usually considered to be transcriptional. In mouse, stimulation by lipopolysaccharide (LPS) or by one of the pro-inflammatory cytokines (e.g., IL-1β, TNFα, IFNγ) is sufficient to induce high amounts of iNOS, whereas in human cells a mixture of several stimuli is needed to achieve iNOS induction (Xie and Nathan, 1994). These species-dependent differences were explained by the many differences found between the human and mouse iNOS promoters (Xie and Nathan, 1994; De Vera et al., 1996). The human promoter is longer and more complex than the mouse promoter, and consists of many binding sites for transcription factors that mediate both enhancement and inhibition of iNOS transcription, such as AP-1, C/EBPβ, EGFR-STAT3, HMGA1, p53, KLF6, five NF-κB sites, Oct-1, two binding sites for IRF-1/STAT-1α, HIF-1, Tcf-4, YY1 and many more (Taylor et al., 1998; Pautz et al., 2010). Only some of these sites can be found in the mouse promoter, which is shorter, and contains proximal and a distal regulatory regions that include mostly NF-κB and IRF-1 binding sites that mediate induction by LPS and IFNγ, respectively. Because of these differences, it was suggested that iNOS effects in mouse tumor models are different than in human tumors, as human cells tend to express lower levels of iNOS and generate less NO (Ambs and Glynn, 2011). However, high amounts of iNOS can be expressed in human cells, provided that a sufficiently strong stimulation is introduced consisting of a mixture of several cytokines *in vitro*, or during inflammation *in vivo* (Xie and Nathan, 1994; Albina, 1995). Furthermore, the hypoxic microenvironment in the tumor dictates a reduced production of NO, regardless of the high expression of the protein (Melillo et al., 1996; Daniliuc et al., 2003). Thus, we maintain that NO concentrations in the tumor are reduced in all species in correlation to the tumor size, indicating that NO production in large, hypoxic tumors is reduced while iNOS protein may be highly expressed in the tumor cells and infiltrating macrophages (Perske et al., 2010). Therefore, the role of iNOS protein expression as a prognostic indicator must be re-examined.

The cytokine network that regulates tumor cell-macrophage interactions is quite complex. In addition to the anti-inflammatory microenvironment (e.g., TGFβ, IL-10, and PGE_2_) that invokes immunosuppression and reprograms macrophage toward M2 activation, pro-inflammatory cytokines (e.g. TNFα, IL-1β and IFNγ) are also present, albeit in relatively low concentrations. At such levels, these cytokines serve to induce adhesion molecules, MMPs, VEGF, and even COX-2 and PGE_2_ production (Dinarello, 2006, 2010). Another microenvironmental factor is the presence of apoptotic cells that release many factors, including shingosine-1-phosphate (S1P) that is taken up by TAMs and repolarizes them toward M2 activation. M2 activated macrophages increase the expression of arginase-I, which changes their iNOS/arginase-I ratio and reduces their ability to produce NO/RNS (Weigert and Brune, 2008). A special role was highlighted for CSF-1, which is secreted from tumor cells and helps recruit macrophages and sustain them in the tumoral microenvironment, and to EGF, which is secreted from the infiltrating macrophages and serves to induce tumor cell migration and invasion (Hernandez et al., 2009). In respect to iNOS regulation, these central mediators also affect its expression, as macrophage EGF induces iNOS in tumor cells (Lo et al., 2005) and tumor cell CSF-1 induces iNOS in macrophages (Lin et al., 2010).

Another important checkpoint is the stability of the iNOS mRNA, which is mediated primarily by the AU-rich elements (ARE) found in the 3′-UTR regions of the transcript. Different RNA binding proteins compete for the binding to the 3′-UTR of iNOS mRNA, including HuR which usually stabilizes mRNAs and is increased upon cytokine induction, and KSRP and tristetraprolin (TTP), which usually mediate destabilization (Pautz et al., 2010). In murine cells, iNOS mRNA degradation was enhanced by TGFβ, and was mediated by the RNA binding proteins PTB (hnRNP I) and hnRNP L (Pautz et al., 2010). Thus, the balance between these proteins may mediate cell type-specific regulation of iNOS expression.

Translation of iNOS protein may be inhibited by small, non-coding RNA molecules known as microRNAs (miRNA). However, there is no direct evidence for the binding of specific miRNAs to iNOS mRNA. One report mentions the indirect translational inhibition of iNOS mRNA through the inhibition of the suppressor of cytokine signal (SOCS-1) mRNA by miR-155 (Wang et al., 2009), and we (Perske et al., 2010) and others (Dai et al., 2008) have shown the involvement of miR-146 in iNOS regulation.

Finally, the activity of the iNOS enzyme is also tightly regulated. Since the enzyme requires L-arginine as its substrate, arginine availability, transport or consumption may have profound implications on iNOS activity. Likewise, mechanisms regulating the availability of additional co-factors, like tetrahydrobiopterin (BH4), also affect iNOS activity (Pautz et al., 2010). The activity of iNOS demands that the protein is homodimerized to ensure correct electron transfer. Protein–protein interactions with additional proteins, such as NAP110 (Ratovitski et al., 1999b) and kalirin (Ratovitski et al., 1999a) were shown to inhibit iNOS activity, whereas other proteins, such as rac2 (Kuncewicz et al., 2001) and α-acitinin-4 (Daniliuc et al., 2003) are required for its activity. The latter two proteins ensure that iNOS is properly localized at the cortical zone, just underneath the plasma membrane, and similar to the other NOS isoforms, enable its activity at this cellular compartment. Disruption of this interaction (e.g., by hypoxia) displaces the enzyme back to the cytoplasm and renders it inactive.

## NO production by tumor cells

Tumor cells, and not only macrophages, can induce iNOS expression and NO production. However, the potential biological relevance of iNOS expression in different malignant human tumors is still controversial, mostly because of technical reasons. Expression of iNOS is often determined by immunohistochemistry, western blot analysis or by real-time RT-PCR—all of which are basically semi-quantitative approaches. Most times, these techniques are applied on paraffin-embedded archival specimens, but these may produce unreliable results due to mRNA degradation in the paraffin-embedded blocks, or due to the recently emerging observations that iNOS protein expression does not necessarily correspond to NO production. Moreover, different ways to score iNOS immune reactivity (e.g., % of positive cells and/or intensity of staining) make comparison of these studies difficult. Measurement of the activity of the protein is thus restricted to fresh tissues, using primarily the indirect Griess reaction to measure accumulation of nitrates and nitrites (Cianchi et al., 2004), or the direct approach of measuring the conversion of L-[^3^H]-arginine to L-[^3^H]-citrulline (Koh et al., 1999; Franchi et al., 2006). Another indirect approach to indicate iNOS activity is the immunohistochemical detection of nitrotyrosinated proteins (Goto et al., 1999; Gochman et al., 2012) or 8-nitro-guanine DNA adducts (Chaiyarit et al., 2005; Ma et al., 2006) that can also be applied on paraffin embedded tumor specimens. However, these indirect approaches do not quantify the extent of iNOS activity, they may be influenced by high activity of other NOS isoforms or by the generation of other RNS (e.g., hypochlorous acid and nitrites that may also nitrotyrosinate proteins) (Radi, 2004), and they are very difficult to compare due to the use of different antibodies or different staining protocols.

Bearing in mind those difficulties, we have tried to critically review the literature, asking whether iNOS expression is correlated with tumoral grade and stage and with poor prognosis, and whether it is limited to macrophages or to tumor cells in specific types of cancer. Table 1 present the conflicting results of this comparison, and emphasizes how poorly understood the role of NO in tumor biology remains.

**Table 1:** **Patterns of iNOS expression and NO production in tumor cells, as influenced by tumor grade and stage**.

**Tumor type**	**Prognosis/survival**	**iNOS activity**	**Expression of iNOS in infiltrating immune cells**	**References**
**EXPRESSION OF INOS INVERSELY CORRELATES WITH TUMOR GRADE AND STAGE OR WITH METASTASES**				
Ovarian cancer	No effect on prognosis; Low iNOS expression correlates with poor prognosis	N/A^a^; Low intra-cystic NO levels in advanced grade	N/M^b^; Strong iNOS staining in macrophages or mononuclear cells	Klimp et al., 2001; Ozel et al., 2006; Anttila et al., 2007; Nomelini et al., 2008
Colorectal cancer	N/M; Low iNOS expression correlates with low survival	N/A; Reduced in tumors relative to normal tissue	Strong staining in mononuclear cells	Moochhala et al., 1996; Ropponen et al., 2000; Hao et al., 2001; Ohta et al., 2006
Breast cancer	N/M	N/A	Strong iNOS staining of macrophages only in grade III tumors	Tschugguel et al., 1999
Brain cancer	No effect	N/A	Some stromal staining of iNOS	Giannopoulou et al., 2006
Lung cancer (NSCLC)	High iNOS expression predicts better survival	N/A	Strong iNOS staining in alveolar macrophages	Puhakka et al., 2003
Cervical cancer	N/M; High iNOS expression correlates with favorable prognosis, low risk for recurrence	N/A	N/M; Some expression in inflammatory infiltrate	Mazibrada et al., 2008; Eggen et al., 2011
**EXPRESSION OF INOS DIRECTLY CORRELATES WITH TUMOR PROGRESSION, GRADE/STAGE, OR METASTASES**				
Malignant melanoma	High iNOS expression is associated with invasiveness, metastases, and increased risk for death. No expression in melanocytic naevi	N/A	N/M, Intense staining of macrophages as tumor progresses	Massi et al., 2001; Ekmekcioglu et al., 2006
Colorectal cancer	N/M; High iNOS expression associated with poor survival	N/A; Increased nitrotyrosine staining	Expression of iNOS in few inflammatory mononuclear cells	Zafirellis et al., 2010; Gochman et al., 2012
Breast cancer	No prognostic effect; Strong iNOS associated with poor prognosis in ER-negative patients or with lower disease-free survival rates	N/A	Strong iNOS staining of stromal cells; No iNOS staining in stromal cells	Vakkala et al., 2000; Bulut et al., 2005; Glynn et al., 2010
Brain cancer	No prognostic effect	N/A	N/M	Hara and Okayasu, 2004
Lung cancer (NSCLC)	High expression relative to no-tumor tissues	Elevated in tumors (Griess)	Few stromal cells may be stained	Lee et al., 2003
Cervical cancer	High iNOS expression is associated with decreased survival and metastases	N/A	N/M	Chen et al., 2005
Gastric cancer	High iNOS expression, especially when accompanied by COX-2 staining, is associated with poor prognosis, invasiveness and/or metastasis	N/A; Increased nitrotyrosine staining	N/M; Weak to moderate positive staining in stromal mononuclear cells	Rajnakova et al., 2001; Feng et al., 2002; Li and Xu, 2005; Chen et al., 2006; Zhang et al., 2011
Head and neck (HNSCC)	N/M; High iNOS expression correlates with metastases and poor prognosis or increased 5-year recurrence rate	N/A; Elevated in carcinoma	Positive iNOS staining in inflammatory cells, probably macrophages	Chen et al., 2002; Franchi et al., 2002; Zhang et al., 2005; Ou Yang et al., 2011
Oral cancer	Expression of iNOS correlated with metastasis	N/A	Positive iNOS staining in stroma cells, probably macrophages	Chen et al., 2002
Pancreatic cancer	High iNOS expression is associated with lymph node metastases	N/A	N/A	Kasper et al., 2004
**NO CORRELATION TO TUMOR GRADE AND STAGE/NOT CONCLUSIVE**				
Cervical cancer	No effect	N/A	N/M; Some positive stromal cell	Oka et al., 2003
Head and neck (HNSCC)	No prognostic effect; iNOS expression is not associated with tumor grade; iNOS activity is associated with lymph node metastasis	N/A; Elevated in tumor periphery	Occasional staining of mononuclear cells; positive staining of macrophages	Pukkila et al., 2002; Jayasurya et al., 2003; Franchi et al., 2006
Bladder cancer	Strong iNOS staining in all bladder tissue, regardless of stage and grade	N/A; No change or elevated nitrites in urine samples from TCC relative to controls	N/M; Strong staining in inflammatory cells (macrophages and neutrophils)	Swana et al., 1999; Eijan et al., 2002; Lin et al., 2003; Sandes et al., 2005
Pancreatic cancer	No prognostic effect	N/A	N/M; iNOS positive stroma cells	Vickers et al., 1999; Kong et al., 2002

a N/A, not assayed.

b N/M, not mentioned.

In certain types of cancer (e.g., gastric cancer, melanoma) increased iNOS expression is found to be associated with tumor stage and grade or with tumor progression toward metastases, as well as with poor prognosis. In contrast, in other types of cancer (e.g., ovarian cancer), iNOS expression is reduced with tumor progression and with poor prognosis. Studies of some tumor types (e.g., colorectal, breast, brain, lung, and cervical cancers) are controversial, indicating either increased or reduced iNOS expression as tumor progresses, whereas in yet other types of cancer (e.g., bladder carcinoma, pancreatic, cervical cancers) positive and even strong iNOS expression was not correlated with either grade/stage or with prognosis. However, in all the studies we found (Table 1), moderate or strong expression of iNOS could be detected in the immunohistochemical images within stromal or inflammatory infiltrating cells, which in some studies were even identified as macrophages. Macrophage iNOS expression, however, was not correlated with prognosis, survival rates, invasiveness or tumor recurrence after therapy.

Evidently, these conflicting results reflect our lack of understanding of the many roles NO plays within the tumor, so that we can only speculate on what may be happening. These results might indicate a different role for iNOS expression in macrophages vs. tumor cells. Macrophages in the tumor stroma exhibit strong iNOS expression regardless of tumor grade and stage, and may produce high levels of NO/RNS that are gradually diminished as they infiltrate the hypoxic core of the tumor. The same is probably true for the tumor cells, and we can assume that tumor cells that are close to the hypoxic core produce less NO/RNS. Thus, the ability of the tumor microenvironment to uncouple iNOS expression and NO production (e.g., via hypoxia) may result in a gradient of NO/RNS concentrations and make it very difficult to assess their true levels within the tumor. The few studies (Table 1) that showed accumulation of nitrotyrosinylated proteins and interpreted these as a measure of increased NO/RNS production are not necessarily right, as protein nitrotyrosinylation is an irreversible reaction that may accumulate over time as the tumor progresses. It is possible that generation of high NO/RNS levels induce genetic instability, not only during the early stages of tumor development, but also as an on-going process, which helps tumor cells accumulate more mutations and further advance to the next malignant stage. It is equally possible that despite the high expression of iNOS protein, the enzyme is rendered inactive, and produces low amounts of NO/RNS that are pro-angiogenic and contribute to tumor aggressiveness. Thus, it is highly important to develop new techniques that will allow to precisely determine NO/RNS concentrations within tumors, preferably in paraffin-embedded archival specimens.

Finally, the fact that such conflicting data are observed in certain cancer types, whereas other cancer types reveal a more consistent behavior, may suggest that other, yet unidentified factors, are involved in the regulation of iNOS activity. Such factors may include components of the specific tissue (e.g., ECM proteins, interstitial cells), or the tumor cells themselves. The fact that macrophages express iNOS in all types of tumors may suggest that tumor cells differently regulate their iNOS expression and NO production.

## Tumor cell production of NO—future perspectives

High levels of NO are strongly associated with initiation of apoptosis, and therefore, it seems reasonable to try and manipulate tumor cells to maintain high levels of NO/RNS concentrations as means of therapeutic intervention. In fact, early studies demonstrated that manipulating tumor cells to produce high NO/RNS inhibited tumor growth. For example, orthotopically implanting pancreatic tumor cell lines that expressed different levels of iNOS showed that tumor cells with low iNOS expression developed pancreatic tumors with metastases to the liver and formed ascites, while tumor cells with high level of iNOS expression did not develop tumors (Wang et al., 2003). In other studies, transfection of tumor cells with the iNOS gene using adenoviral or retroviral vectors lead to their ability to produce NO and other pro-angiogenic proteins, but these cells did not form tumors in nude mice due to initiation of apoptosis (Le et al., 2005) or developed small tumors with no lung metastases in comparison to non-transfected cells (Juang et al., 1998). These studies highlighted the importance of tumor cell iNOS expression, but did not take into account the effects of the infiltrating macrophages or the changing microenvironment. Furthermore, since iNOS was continuously overexpressed in the tumor cells, it is likely that their apoptotic death occurred at an early stage of tumor development, before macrophages were recruited and “re-educated” to become pro-angiogenic and immunosuppressive. Moreover, such a manipulation of tumor cells that involves their transfection with an iNOS construct designed to cause high iNOS expression is clearly not easily feasible in the clinical real-life scenario, where tumors are often diagnosed after they have gained considerable mass and created an immunosuppressive microenvironment.

A different approach to treat tumors with NO/RNS was to use macrophages. Macrophages were isolated from a patient, activated *ex vivo* as M1 macrophages, and then re-introduced back to the same patient. Three qualities make this autologous macrophage adoptive transfer an appealing approach: (1) their tumoricidial abilities that is based on production of high concentration of cytotoxic molecules such as NO/RNS; (2) the ease to isolate them from patients in large numbers and to activate them *ex vivo* before their re-infusion; (3) their ability to home directly to the tumor, thereby specifically targeting the tumor cells (Murdoch et al., 2004; Allavena et al., 2008).

However, previous experiments performed on human subjects, where monocytes were collected, classically stimulated *ex vivo* with IFNγ and/or LPS, and autologously re-infused into the patient, proved that although the process was safe with only minor side effects, no significant beneficial clinical effects were observed (Andreesen et al., 1998; Hennemann et al., 1998). Another study showed that autologous IFNγ-activated macrophages that were intrapleurally injected into patients suffering from malignant mesothelioma showed only limited and insignificant (about 14%) anti-tumor response (Monnet et al., 2002), although these macrophages produced high levels of TNFα and NO/RNS and proved to be cytotoxic to tumor cells *in vitro*. In mice, such treatment resulted in inhibition of metastasis formation, with sometimes attenuated growth, but no regression of the primary tumor (Andreesen et al., 1998; Perske et al., 2010). Adoptive transfer of activated macrophages that were first transduced with macrophage colony stimulating factor (M-CSF) and IFNγ by recombinant adenovirus infection and were tumor-pulsed prior to their re-infusion, succeeded in reducing pulmonary metastases in a B16F10 melanoma model. These gene-modulated macrophages exhibited increased secretion of cytotoxic molecules, including NO, and increased antigen presentation when pulsed with tumor lysates, suggesting that on-going activation of macrophages *in vivo* is critical to their anti-tumor effects and to their ability to recruit specific cytotoxic T cells (Lei et al., 2000). However, such an approach, which demands isolation of macrophages in large amounts followed by their gene-modulation in combination with isolation of enough tumor tissue to produce lysates for macrophage pulsing, seems very elaborate and difficult to achieve in humans.

In retrospect, the macrophage therapy approach probably failed to take into account the ability of the hypoxic and immunosuppressive microenvironment to skew the *ex vivo* M1-activated macrophages back toward an M2 mode of activation, which resulted in failure of these trials. The tumor microenvironment, which is rich in anti-inflammatory mediators (e.g., TGFβ, IL-10, PGE_2_) and with apoptotic cell debris, directly neutralizes such pre-treated M1-activated macrophages (Kees and Egeblad, 2011). Specifically, even if such macrophages expressed high levels of the iNOS protein, the hypoxic microenvironment would inhibit their production of high amounts of NO/RNS. Additionally, macrophage therapy approach failed to provide on-going signals that would maintain the anti-tumoral phenotype of the infused macrophages.

How can we, then, manipulate tumor cell-macrophage interactions in order to eradicate the tumor? It is still advisable to use macrophages, but only as long as we can maintain their skew toward M1 activation. It is possible that after surgery, radio- or chemotherapy, when the tumor mass is reduced, regulatory cells (e.g., Treg or MDSCs) are diminished, and the microenvironment is less hypoxic and immunosuppressive, thus generating a small window of opportunity for a more successful macrophage therapy. Indeed, attempts to combine such therapies and activate the innate immune cells in a timely manner are now beginning to be explored (Kees and Egeblad, 2011).

We can also use NO as a radio- or chemo-sensitizer to enhance the beneficial effects or radio- and chemotherapy. It has been shown that well-oxygenated tumor cells that reside near blood vessels or at the tumor rim are radiosensitive, whereas those that are located in hypoxic areas may be 3-times more radio-resistant. Irradiation kills proliferating tumor cells through accumulation of DNA damage that is dependent on presence of oxygen and the free radicals it generates. Hypoxia is believed to increase radio-resistance through the accumulation of HIF-1, which in turn, down-regulates pro-apoptotic genes, enhances multidrug resistant proteins and induces expression of genes like VEGF and enzymes of the glycolytic pathway, thus ensuring blood supply and energy required for tumor cell survival and proliferation (Fitzpatrick et al., 2008; Yasuda, 2008). The potential use of NO as a radio- and chemo-sensitizer for such resistant tumor cells is currently being explored, and several mechanisms could explain its effects. By binding to cytochrome c oxidase, NO can inhibit mitochondrial respiration and generate ROS that activate PHDs and HIF-1 hydroxylation, leading to increased degradation of HIF-1 in hypoxia (Fitzpatrick et al., 2008; Yasuda, 2008). Inhibition of the mitochondria also diverts oxygen from this organelle to the cytoplasm, thus protecting cells from death. Much like oxygen, NO can directly damage DNA, lipids and proteins (probably though generation of peroxynitrite), and systemically NO has a vasodilative effect that provides more blood supply to the tumor cells and maintain their oxygenation. Thus, NO or NO-donors have been explored as potential adjuvants for radiotherapy. However, results remain controversial, and studies show both beneficial and detrimental effects, depending on the tumor microenvironment, NO concentrations, the oxygenated state of the tumor, systemic responses and more (Oronsky et al., 2012). Use of NO-donors to radio-sensitize tumor cells may also cause serious systemic side effects, such as hypotension, which may result in further increasing tumor hypoxia and tumor cell radio-resistance, and the use of IFNγ administration to induce iNOS expression is limited because of its toxicity and vascular effects (Fitzpatrick et al., 2008). However, this highlights again the importance of endogenous NO production by the tumor cells.

NO/RNS production plays a key role in tumor cell-macrophage interactions, as both cell types can produce it. Table 1 demonstrates that in some tumor types, high grade tumors or metastatic tumor cells tend to reduce their iNOS protein expression or lose it completely, as a means of escaping the immune system. We have previously shown in a murine renal cell carcinoma (RENCA) model injected subcutaneously, that even high concentrations of NO/RNS within the tumor *in vivo*, whether delivered by an NO donor (NOC-18) or by M1-activated macrophages, could only attenuate tumor growth, but did not regress the tumor (Perske et al., 2010). Furthermore, *in vitro* co-culture of RENCA tumor cells that did not express iNOS with RAW 264.7 macrophages, in the presence of IFNγ and LPS that strongly induced macrophage iNOS expression, did not result in tumor cell death. Only when these tumor cells were induced to express iNOS, even in low levels, by alleviating the translational inhibition on the protein through neutralization of microRNA-146a (miR-146a), macrophage-induced tumor cell death was initiated (Perske et al., 2010). Thus, high exogenous concentrations of NO/RNS in the tumor microenvironment are not sufficient to kill tumor cells, and the decision whether tumor cells will undergo apoptosis depends on their own ability to produce NO. Other studies that demonstrated the importance of endogenous tumor cell NO production to their susceptibility to apoptosis support our findings (Le et al., 2005). Different tumor cells were transfected with wild type or mutant iNOS constructs that resulted in different degrees of iNOS activity, and then implanted *s.c.* into nude mice. NO production in the wild type cells strongly suppressed tumor cell proliferation and tumor growth by inducing their apoptosis in a concentration-dependent way, whereas induction of the expression of pro-angiogenic factors, such as VEGF and IL-8 remained constant (Le et al., 2005). These findings and our own, highlight one strategy that tumor cells may take to evade macrophage-induced death by reducing or abrogating their iNOS expression. It is possible that miRNA-146a affects additional targets besides iNOS, and thus acts as a general stimulator, this time of the tumor cells rather than the macrophages. These findings also highlight the importance of the dialogue between tumor cells and macrophages, and underscore the degree of control that tumor cells exert over their environment and the functioning of infiltrating cells.

This current understanding of the important translational regulation of iNOS expression through miRNA-146a, that allows tumor cells to evade macrophage-induced death, may be expanded to envision new therapeutic approaches that are based on the ability to manipulate NO production in the tumor cells. To do this, we must first better understand the precise machinery that allows miR-146a to inhibit iNOS translation, and then find an efficient delivery system of anti-miR-146a specifically into tumor cells, so we can manipulate iNOS production in these cells. Such manipulation of iNOS expression in tumor cells, combined with infusion of *ex vivo* M1-activated macrophages could become an attractive therapeutic approach, which overrides both the immunosuppressive effects of the microenvironment and the evasion strategy of tumor cells.

In conclusion, it is the overall concentrations of NO/RNS, rather than the extent of iNOS expression, that ultimately determine their activities. Low levels of NO/RNS are pro-angiogenic and support immune evasion, whereas high amounts trigger apoptosis. Thus, our goal is to increase NO/RNS production in both tumor cells and macrophages, by overcoming their respective inhibitory mechanisms, so that the pro-angiogenic effects of NO/RNS are inhibited, the immune system regains recognition of the tumor cells and its pro-apoptotic effects are enhanced to effectively eradicate the tumor.

### Conflict of interest statement

The authors declare that the research was conducted in the absence of any commercial or financial relationships that could be construed as a potential conflict of interest.

## References

[B1] Aktan F. (2004). iNOS-mediated nitric oxide production and its regulation. Life Sci. 75, 639–653 10.1016/j.lfs.2003.10.042 15172174

[B2] Albina J. E. (1995). On the expression of nitric oxide synthase by human macrophages. Why no NO? J. Leukoc. Biol. 58, 643–649 7499961 10.1002/jlb.58.6.643

[B3] Alderton W. K. Cooper C. E. Knowles R. G. (2001). Nitric oxide synthases: structure, function and inhibition. Biochem. J. 357, 593–615 10.1042/0264-6021:3570593 11463332 PMC1221991

[B4] Allavena P. Sica A. Garlanda C. Mantovani A. (2008). The Yin-Yang of tumor-associated macrophages in neoplastic progression and immune surveillance. Immunol. Rev. 222, 155–161 10.1111/j.1600-065X.2008.00607.x 18364000

[B5] Ambs S. Glynn S. A. (2011). Candidate pathways linking inducible nitric oxide synthase to a basal-like transcription pattern and tumor progression in human breast cancer. Cell Cycle 10, 619–624 10.4161/cc.10.41.4864 21293193 PMC3043082

[B6] Andreesen R. Hennemann B. Krause S. W. (1998). Adoptive immunotherapy of cancer using monocyte-derived macrophages: rationale, current status, and perspectives. J. Leukoc. Biol. 64, 419–426 9766621 10.1002/jlb.64.4.419

[B7] Anttila M. A. Voutilainen K. Merivalo S. Saarikoski S. Kosma V. M. (2007). Prognostic significance of iNOS in epithelial ovarian cancer. Gynecol. Oncol. 105, 97–103 10.1016/j.ygyno.2006.10.049 17174383

[B8] Black S. M. Devol J. M. Wedgwood S. (2008). Regulation of fibroblast growth factor-2 expression in pulmonary arterial smooth muscle cells involves increased reactive oxygen species generation. Am. J. Physiol. Cell Physiol. 294, C345–C354 10.1152/ajpcell.00216.2007 17942638 PMC3970933

[B9] Bronte V. (2009). Myeloid-derived suppressor cells in inflammation: uncovering cell subsets with enhanced immunosuppressive functions. Eur. J. Immunol. 39, 2670–2672 10.1002/eji.200939892 19757440

[B10] Brune B. (2003). Nitric oxide: NO apoptosis or turning it ON? Cell Death Differ. 10, 864–869 10.1038/sj.cdd.4401261 12867993

[B11] Bui J. D. Schreiber R. D. (2007). Cancer immunosurveillance, immunoediting and inflammation: independent or interdependent processes? Curr. Opin. Immunol. 19, 203–208 10.1016/j.coi.2007.02.001 17292599

[B12] Bulut A. S. Erden E. Sak S. D. Doruk H. Kursun N. Dincol D. (2005). Significance of inducible nitric oxide synthase expression in benign and malignant breast epithelium: an immunohistochemical study of 151 cases. Virchows Arch. 447, 24–30 10.1007/s00428-005-1250-2 15947943

[B13] Chaiyarit P. Ma N. Hiraku Y. Pinlaor S. Yongvanit P. Jintakanon D. (2005). Nitrative and oxidative DNA damage in oral lichen planus in relation to human oral carcinogenesis. Cancer Sci. 96, 553–559 10.1111/j.1349-7006.2005.00096.x 16128740 PMC11160045

[B14] Chen C. N. Hsieh F. J. Cheng Y. M. Chang K. J. Lee P. H. (2006). Expression of inducible nitric oxide synthase and cyclooxygenase-2 in angiogenesis and clinical outcome of human gastric cancer. J. Surg. Oncol. 94, 226–233 10.1002/jso.20372 16900533

[B15] Chen H. H. Su W. C. Chou C. Y. Guo H. R. Ho S. Y. Que J. (2005). Increased expression of nitric oxide synthase and cyclooxygenase-2 is associated with poor survival in cervical cancer treated with radiotherapy. Int. J. Radiat. Oncol. Biol. Phys. 63, 1093–1100 10.1016/j.ijrobp.2005.03.062 16099602

[B16] Chen Y. K. Hsue S. S. Lin L. M. (2002). Increased expression of inducible nitric oxide synthase for human buccal squamous-cell carcinomas: immunohistochemical, reverse transcription-polymerase chain reaction (RT-PCR) and *in situ* RT-PCR studies. Head Neck 24, 925–932 10.1002/hed.10131 12369071

[B17] Chowdhury R. Godoy L. C. Thiantanawat A. Trudel L. J. Deen W. M. Wogan G. N. (2012). Nitric oxide produced endogenously is responsible for hypoxia-induced HIF-1alpha stabilization in colon carcinoma cells. Chem. Res. Toxicol. 25, 2194–2202 10.1021/tx300274a 22971010 PMC3472092

[B18] Cianchi F. Cortesini C. Fantappie O. Messerini L. Sardi I. Lasagna N. (2004). Cyclooxygenase-2 activation mediates the proangiogenic effect of nitric oxide in colorectal cancer. Clin. Cancer Res. 10, 2694–2704 10.1158/1078-0432.CCR-03-0192 15102673

[B19] Coffelt S. B. Chen Y. Y. Muthana M. Welford A. F. Tal A. O. Scholz A. (2011). Angiopoietin 2 stimulates TIE2-expressing monocytes to suppress T cell activation and to promote regulatory T cell expansion. J. Immunol. 186, 4183–4190 10.4049/jimmunol.1002802 21368233

[B20] Coffelt S. B. Hughes R. Lewis C. E. (2009). Tumor-associated macrophages: effectors of angiogenesis and tumor progression. Biochim. Biophys. Acta 1796, 11–18 10.1016/j.bbcan.2009.02.004 19269310

[B21] Condeelis J. Pollard J. W. (2006). Macrophages: obligate partners for tumor cell migration, invasion, and metastasis. Cell 124, 263–266 10.1016/j.cell.2006.01.007 16439202

[B22] Corzo C. A. Condamine T. Lu L. Cotter M. J. Youn J. I. Cheng P. (2010). HIF-1alpha regulates function and differentiation of myeloid-derived suppressor cells in the tumor microenvironment. J. Exp. Med. 207, 2439–2453 10.1084/jem.20100587 20876310 PMC2964584

[B23] Dai R. Phillips R. A. Zhang Y. Khan D. Crasta O. Ahmed S. A. (2008). Suppression of LPS-induced Interferon-gamma and nitric oxide in splenic lymphocytes by select estrogen-regulated microRNAs: a novel mechanism of immune modulation. Blood 112, 4591–4597 10.1182/blood-2008-04-152488 18791161 PMC2597130

[B24] Daniliuc S. Bitterman H. Rahat M. A. Kinarty A. Rosenzweig D. Lahat N. (2003). Hypoxia inactivates inducible nitric oxide synthase in mouse macrophages by disrupting its interaction with alpha-actinin 4. J. Immunol. 171, 3225–3232 12960352 10.4049/jimmunol.171.6.3225

[B25] De Palma M. Murdoch C. Venneri M. A. Naldini L. Lewis C. E. (2007). Tie2-expressing monocytes: regulation of tumor angiogenesis and therapeutic implications. Trends Immunol. 28, 519–524 10.1016/j.it.2007.09.004 17981504

[B26] De Palma M. Venneri M. A. Galli R. Sergi Sergi L. Politi L. S. Sampaolesi M. (2005). Tie2 identifies a hematopoietic lineage of proangiogenic monocytes required for tumor vessel formation and a mesenchymal population of pericyte progenitors. Cancer Cell 8, 211–226 10.1016/j.ccr.2005.08.002 16169466

[B27] De Vera M. E. Shapiro R. A. Nussler A. K. Mudgett J. S. Simmons R. L. Morris S. M. (1996). Transcriptional regulation of human inducible nitric oxide synthase (NOS2) gene by cytokines: initial analysis of the human NOS2 promoter. Proc. Natl. Acad. Sci. U.S.A. 93, 1054–1059 10.1073/pnas.93.3.1054 8577713 PMC40029

[B28] Dinarello C. A. (2006). The paradox of pro-inflammatory cytokines in cancer. Cancer Metastasis Rev. 25, 307–313 10.1007/s10555-006-9000-8 17029030

[B29] Dinarello C. A. (2010). Why not treat human cancer with interleukin-1 blockade? Cancer Metastasis Rev. 29, 317–329 10.1007/s10555-010-9229-0 20422276 PMC2865633

[B30] Donzelli S. Switzer C. H. Thomas D. D. Ridnour L. A. Espey M. G. Isenberg J. S. (2006). The activation of metabolites of nitric oxide synthase by metals is both redox and oxygen dependent: a new feature of nitrogen oxide signaling. Antioxid. Redox Signal. 8, 1363–1371 10.1089/ars.2006.8.1363 16910783

[B31] Dulak J. Jozkowicz A. (2003). Regulation of vascular endothelial growth factor synthesis by nitric oxide: facts and controversies. Antioxid. Redox Signal. 5, 123–132 10.1089/152308603321223612 12626124

[B32] Dunn G. P. Old L. J. Schreiber R. D. (2004). The three Es of cancer immunoediting. Annu. Rev. Immunol. 22, 329–360 10.1146/annurev.immunol.22.012703.104803 15032581

[B33] Eggen T. Sager G. Arnes M. Pettersen I. Orbo A. (2011). Expression of iNOS–a favourable prognostic marker for early-stage carcinoma of the uterine cervix. Anticancer Res. 31, 2319–2325 21737658

[B34] Eijan A. M. Piccardo I. Riveros M. D. Sandes E. O. Porcella H. Jasnis M. A. (2002). Nitric oxide in patients with transitional bladder cancer. J. Surg. Oncol. 81, 203–208 10.1002/jso.10170 12451625

[B35] Ekmekcioglu S. Ellerhorst J. A. Prieto V. G. Johnson M. M. Broemeling L. D. Grimm E. A. (2006). Tumor iNOS predicts poor survival for stage III melanoma patients. Int. J. Cancer 119, 861–866 10.1002/ijc.21767 16557582

[B36] Feng C. W. Wang L. D. Jiao L. H. Liu B. Zheng S. Xie X. J. (2002). Expression of p53, inducible nitric oxide synthase and vascular endothelial growth factor in gastric precancerous and cancerous lesions: correlation with clinical features. BMC Cancer 2:8 10.1186/1471-2407-2-8 11978184 PMC113262

[B37] Fitzpatrick B. Mehibel M. Cowen R. L. Stratford I. J. (2008). iNOS as a therapeutic target for treatment of human tumors. Nitric Oxide 19, 217–224 10.1016/j.niox.2008.05.001 18515106

[B38] Franchi A. Gallo O. Paglierani M. Sardi I. Magnelli L. Masini E. (2002). Inducible nitric oxide synthase expression in laryngeal neoplasia: correlation with angiogenesis. Head Neck 24, 16–23 10.1002/hed.10045 11774398

[B39] Franchi A. Massi D. Santucci M. Masini E. Rossi Degl'innocenti D. Magnelli L. (2006). Inducible nitric oxide synthase activity correlates with lymphangiogenesis and vascular endothelial growth factor-C expression in head and neck squamous cell carcinoma. J. Pathol. 208, 439–445 10.1002/path.1892 16278821

[B40] Gabrilovich D. I. Nagaraj S. (2009). Myeloid-derived suppressor cells as regulators of the immune system. Nat. Rev. Immunol. 9, 162–174 10.1038/nri2506 19197294 PMC2828349

[B41] Giannopoulou E. Ravazoula P. Kalofonos H. Makatsoris T. Kardamakis D. (2006). Expression of HIF-1alpha and iNOS in astrocytic gliomas: a clinicopathological study. In Vivo 20, 421–425 16724682

[B42] Glynn S. A. Boersma B. J. Dorsey T. H. Yi M. Yfantis H. G. Ridnour L. A. (2010). Increased NOS2 predicts poor survival in estrogen receptor-negative breast cancer patients. J. Clin. Invest. 120, 3843–3854 10.1172/JCI42059 20978357 PMC2964971

[B43] Gochman E. Mahajna J. Shenzer P. Dahan A. Blatt A. Elyakim R. (2012). The expression of iNOS and nitrotyrosine in colitis and colon cancer in humans. Acta Histochem. 114, 827–835 10.1016/j.acthis.2012.02.004 22417974

[B44] Gordon S. Martinez F. O. (2010). Alternative activation of macrophages: mechanism and functions. Immunity 32, 593–604 10.1016/j.immuni.2010.05.007 20510870

[B45] Goto T. Haruma K. Kitadai Y. Ito M. Yoshihara M. Sumii K. (1999). Enhanced expression of inducible nitric oxide synthase and nitrotyrosine in gastric mucosa of gastric cancer patients. Clin. Cancer Res. 5, 1411–1415 10389926

[B46] Hanahan D. Weinberg R. A. (2011). Hallmarks of cancer: the next generation. Cell 144, 646–674 10.1016/j.cell.2011.02.013 21376230

[B47] Hao X. P. Pretlow T. G. Rao J. S. Pretlow T. P. (2001). Inducible nitric oxide synthase (iNOS) is expressed similarly in multiple aberrant crypt foci and colorectal tumors from the same patients. Cancer Res. 61, 419–422 11212223

[B48] Hara A. Okayasu I. (2004). Cyclooxygenase-2 and inducible nitric oxide synthase expression in human astrocytic gliomas: correlation with angiogenesis and prognostic significance. Acta Neuropathol. 108, 43–48 10.1007/s00401-004-0860-0 15088099

[B49] Heller A. (2008). Apoptosis-inducing high (.)NO concentrations are not sustained either in nascent or in developed cancers. Chem. Med. Chem. 3, 1493–1499 10.1002/cmdc.200800257 18759245

[B50] Hennemann B. Beckmann G. Eichelmann A. Rehm A. Andreesen R. (1998). Phase I trial of adoptive immunotherapy of cancer patients using monocyte-derived macrophages activated with interferon gamma and lipopolysaccharide. Cancer Immunol. Immunother. 45, 250–256 10.1007/PL00006671 9439648 PMC11037564

[B51] Hernandez L. Smirnova T. Kedrin D. Wyckoff J. Zhu L. Stanley E. R. (2009). The EGF/CSF-1 paracrine invasion loop can be triggered by heregulin beta1 and CXCL12. Cancer Res. 69, 3221–3227 10.1158/0008-5472.CAN-08-2871 19293185 PMC2820720

[B52] Hollborn M. Stathopoulos C. Steffen A. Wiedemann P. Kohen L. Bringmann A. (2007). Positive feedback regulation between MMP-9 and VEGF in human RPE cells. Invest. Ophthalmol. Vis. Sci. 48, 4360–4367 10.1167/iovs.06-1234 17724228

[B53] Hussain S. P. He P. Subleski J. Hofseth L. J. Trivers G. E. Mechanic L. (2008). Nitric oxide is a key component in inflammation-accelerated tumorigenesis. Cancer Res. 68, 7130–7136 10.1158/0008-5472.CAN-08-0410 18757428 PMC2576291

[B54] Iyer A. K. Azad N. Wang L. Rojanasakul Y. (2008). Role of S-nitrosylation in apoptosis resistance and carcinogenesis. Nitric Oxide 19, 146–151 10.1016/j.niox.2008.04.019 18474261 PMC3226739

[B55] Jayaraman P. Parikh F. Lopez-Rivera E. Hailemichael Y. Clark A. Ma G. (2012). Tumor-expressed inducible nitric oxide synthase controls induction of functional myeloid-derived suppressor cells through modulation of vascular endothelial growth factor release. J. Immunol. 188, 5365–5376 10.4049/jimmunol.1103553 22529296 PMC3358566

[B56] Jayasurya A. Dheen S. T. Yap W. M. Tan N. G. Ng Y. K. Bay B. H. (2003). Inducible nitric oxide synthase and bcl-2 expression in nasopharyngeal cancer: correlation with outcome of patients after radiotherapy. Int. J. Radiat. Oncol. Biol. Phys. 56, 837–845 10.1016/S0360-3016(03)00122-6 12788193

[B57] Jeannin J. F. Leon L. Cortier M. Sassi N. Paul C. Bettaieb A. (2008). Nitric oxide-induced resistance or sensitization to death in tumor cells. Nitric Oxide 19, 158–163 10.1016/j.niox.2008.04.024 18495079

[B58] Jia W. Jackson-Cook C. Graf M. R. (2010). Tumor-infiltrating, myeloid-derived suppressor cells inhibit T cell activity by nitric oxide production in an intracranial rat glioma + vaccination model. J. Neuroimmunol. 223, 20–30 10.1016/j.jneuroim.2010.03.011 20452681 PMC2883008

[B59] Juang S. H. Xie K. Xu L. Shi Q. Wang Y. Yoneda J. (1998). Suppression of tumorigenicity and metastasis of human renal carcinoma cells by infection with retroviral vectors harboring the murine inducible nitric oxide synthase gene. Hum. Gene Ther. 9, 845–854 10.1089/hum.1998.9.6-845 9581907

[B60] Kasper H. U. Wolf H. Drebber U. Wolf H. K. Kern M. A. (2004). Expression of inducible nitric oxide synthase and cyclooxygenase-2 in pancreatic adenocarcinoma: correlation with microvessel density. World J. Gastroenterol. 10, 1918–1922 15222037 10.3748/wjg.v10.i13.1918 PMC4572231

[B61] Kees T. Egeblad M. (2011). Innate immune cells in breast cancer–from villains to heroes? J. Mammary Gland Biol. Neoplasia 16, 189–203 10.1007/s10911-011-9224-2 21789554

[B62] Kimura H. Weisz A. Kurashima Y. Hashimoto K. Ogura T. D'acquisto F. (2000). Hypoxia response element of the human vascular endothelial growth factor gene mediates transcriptional regulation by nitric oxide: control of hypoxia-inducible factor-1 activity by nitric oxide. Blood 95, 189–197 10607702

[B63] Kimura H. Weisz A. Ogura T. Hitomi Y. Kurashima Y. Hashimoto K. (2001). Identification of hypoxia-inducible factor 1 ancillary sequence and its function in vascular endothelial growth factor gene induction by hypoxia and nitric oxide. J. Biol. Chem. 276, 2292–2298 10.1074/jbc.M008398200 11056166

[B64] Klimp A. H. Hollema H. Kempinga C. Van Der Zee A. G. De Vries E. G. Daemen T. (2001). Expression of cyclooxygenase-2 and inducible nitric oxide synthase in human ovarian tumors and tumor-associated macrophages. Cancer Res. 61, 7305–7309 11585770

[B65] Koh E. Noh S. H. Lee Y. D. Lee H. Y. Han J. W. Lee H. W. (1999). Differential expression of nitric oxide synthase in human stomach cancer. Cancer Lett. 146, 173–180 10.1016/S0304-3835(99)00265-7 10656623

[B66] Kong G. Kim E. K. Kim W. S. Lee K. T. Lee Y. W. Lee J. K. (2002). Role of cyclooxygenase-2 and inducible nitric oxide synthase in pancreatic cancer. J. Gastroenterol. Hepatol. 17, 914–921 10.1046/j.1440-1746.2002.02829.x 12164968

[B67] Kuncewicz T. Balakrishnan P. Snuggs M. B. Kone B. C. (2001). Specific association of nitric oxide synthase-2 with Rac isoforms in activated murine macrophages. Am. J. Physiol. Renal Physiol. 281, F326–F336 11457725 10.1152/ajprenal.2001.281.2.F326

[B68] Kundu J. K. Surh Y. J. (2008). Inflammation: gearing the journey to cancer. Mutat. Res. 659, 15–30 10.1016/j.mrrev.2008.03.002 18485806

[B69] Lahat N. Bitterman H. Engelmayer-Goren M. Rosenzweig D. Weiss-Cerem L. Rahat M. A. (2011). Reduced TIMP-2 in hypoxia enhances angiogenesis. Am. J. Physiol. Cell Physiol. 300, C557–C566 10.1152/ajpcell.00177.2010 21148412

[B70] Lala P. K. Chakraborty C. (2001). Role of nitric oxide in carcinogenesis and tumour progression. Lancet Oncol. 2, 149–156 10.1016/S1470-2045(00)00256-4 11902565

[B71] Lancaster J. R. Jr. Xie K. (2006). Tumors face NO problems? Cancer Res. 66, 6459–6462 10.1158/0008-5472.CAN-05-2900 16818612

[B72] Le X. Wei D. Huang S. Lancaster J. R. Jr. Xie K. (2005). Nitric oxide synthase II suppresses the growth and metastasis of human cancer regardless of its up-regulation of protumor factors. Proc. Natl. Acad. Sci. U.S.A. 102, 8758–8763 10.1073/pnas.0409581102 15939886 PMC1150810

[B73] Lechner M. Lirk P. Rieder J. (2005). Inducible nitric oxide synthase (iNOS) in tumor biology: the two sides of the same coin. Semin. Cancer Biol. 15, 277–289 10.1016/j.semcancer.2005.04.004 15914026

[B74] Lee T. W. Chen G. G. Xu H. Yip J. H. Chak E. C. Mok T. S. (2003). Differential expression of inducible nitric oxide synthase and peroxisome proliferator-activated receptor gamma in non-small cell lung carcinoma. Eur. J. Cancer 39, 1296–1301 10.1016/S0959-8049(02)00733-5 12763220

[B75] Lei H. Ju D. W. Yu Y. Tao Q. Chen G. Gu S. (2000). Induction of potent antitumor response by vaccination with tumor lysate-pulsed macrophages engineered to secrete macrophage colony-stimulating factor and interferon-gamma. Gene Ther. 7, 707–713 10.1038/sj.gt.3301162 10800095

[B76] Leon L. Jeannin J. F. Bettaieb A. (2008). Post-translational modifications induced by nitric oxide (NO): implication in cancer cells apoptosis. Nitric Oxide 19, 77–83 10.1016/j.niox.2008.04.014 18474258

[B77] Lewis C. E. Pollard J. W. (2006). Distinct role of macrophages in different tumor microenvironments. Cancer Res. 66, 605–612 10.1158/0008-5472.CAN-05-4005 16423985

[B78] Li L. G. Xu H. M. (2005). Inducible nitric oxide synthase, nitrotyrosine and apoptosis in gastric adenocarcinomas and their correlation with a poor survival. World J. Gastroenterol. 11, 2539–2544 15849807 10.3748/wjg.v11.i17.2539 PMC4305739

[B79] Lin C. W. Shen S. C. Ko C. H. Lin H. Y. Chen Y. C. (2010). Reciprocal activation of macrophages and breast carcinoma cells by nitric oxide and colony-stimulating factor-1. Carcinogenesis 31, 2039–2048 10.1093/carcin/bgq172 20876703

[B80] Lin Z. Chen S. Ye C. Zhu S. (2003). Nitric oxide synthase expression in human bladder cancer and its relation to angiogenesis. Urol. Res. 31, 232–235 10.1007/s00240-003-0302-9 12937869

[B81] Lo H. W. Hsu S. C. Ali-Seyed M. Gunduz M. Xia W. Wei Y. (2005). Nuclear interaction of EGFR and STAT3 in the activation of the iNOS/NO pathway. Cancer Cell 7, 575–589 10.1016/j.ccr.2005.05.007 15950906

[B82] Ma N. Tagawa T. Hiraku Y. Murata M. Ding X. Kawanishi S. (2006). 8-Nitroguanine formation in oral leukoplakia, a premalignant lesion. Nitric Oxide 14, 137–143 10.1016/j.niox.2005.09.012 16290060

[B83] Mantovani A. Allavena P. Sica A. Balkwill F. (2008). Cancer-related inflammation. Nature 454, 436–444 10.1038/nature07205 18650914

[B84] Martinez F. O. Helming L. Gordon S. (2009). Alternative activation of macrophages: an immunologic functional perspective. Annu. Rev. Immunol. 27, 451–483 10.1146/annurev.immunol.021908.132532 19105661

[B85] Massi D. Franchi A. Sardi I. Magnelli L. Paglierani M. Borgognoni L. (2001). Inducible nitric oxide synthase expression in benign and malignant cutaneous melanocytic lesions. J. Pathol. 194, 194–200 10.1002/1096-9896(200106)194:2<194::AID-PATH851>3.0.CO;2-S 11400148

[B86] Mazibrada J. Ritta M. Mondini M. De Andrea M. Azzimonti B. Borgogna C. (2008). Interaction between inflammation and angiogenesis during different stages of cervical carcinogenesis. Gynecol. Oncol. 108, 112–120 10.1016/j.ygyno.2007.08.095 17936343

[B87] Melillo G. Taylor L. S. Brooks A. Cox G. W. Varesio L. (1996). Regulation of inducible nitric oxide synthase expression in IFN-gamma-treated murine macrophages cultured under hypoxic conditions. J. Immunol. 157, 2638–2644 8805668

[B88] Monnet I. Breau J. L. Moro D. Lena H. Eymard J. C. Menard O. (2002). Intrapleural infusion of activated macrophages and gamma-interferon in malignant pleural mesothelioma: a phase II study. Chest 121, 1921–1927 10.1378/chest.121.6.1921 12065358

[B89] Moochhala S. Chhatwal V. J. Chan S. T. Ngoi S. S. Chia Y. W. Rauff A. (1996). Nitric oxide synthase activity and expression in human colorectal cancer. Carcinogenesis 17, 1171–1174 10.1093/carcin/17.5.1171 8640931

[B90] Mosser D. M. (2003). The many faces of macrophage activation. J. Leukoc. Biol. 73, 209–212 10.1189/jlb.0602325 12554797

[B91] Mosser D. M. Edwards J. P. (2008). Exploring the full spectrum of macrophage activation. Nat. Rev. Immunol. 8, 958–969 10.1038/nri2448 19029990 PMC2724991

[B92] Mosser D. M. Zhang X. (2008). Activation of murine macrophages. Curr. Protoc. Immunol. Chapter 14:Unit 14.12. 10.1002/0471142735.im1402s83 PMC2822273 19016446

[B93] Murdoch C. Giannoudis A. Lewis C. E. (2004). Mechanisms regulating the recruitment of macrophages into hypoxic areas of tumors and other ischemic tissues. Blood 104, 2224–2234 10.1182/blood-2004-03-1109 15231578

[B94] Murdoch C. Muthana M. Coffelt S. B. Lewis C. E. (2008). The role of myeloid cells in the promotion of tumour angiogenesis. Nat. Rev. Cancer 8, 618–631 10.1038/nrc2444 18633355

[B95] Nagaraj S. Gabrilovich D. I. (2008). Tumor escape mechanism governed by myeloid-derived suppressor cells. Cancer Res. 68, 2561–2563 10.1158/0008-5472.CAN-07-6229 18413722

[B96] Nagaraj S. Gupta K. Pisarev V. Kinarsky L. Sherman S. Kang L. (2007). Altered recognition of antigen is a mechanism of CD8+ T cell tolerance in cancer. Nat. Med. 13, 828–835 10.1038/nm1609 17603493 PMC2135607

[B97] Nizet V. Johnson R. S. (2009). Interdependence of hypoxic and innate immune responses. Nat. Rev. Immunol. 9, 609–617 10.1038/nri2607 19704417 PMC4343208

[B98] Nomelini R. S. De Abreu Ribeiro L. C. Tavares-Murta B. M. Adad S. J. Murta E. F. (2008). Production of nitric oxide and expression of inducible nitric oxide synthase in ovarian cystic tumors. Mediators Inflamm. 2008:186584 10.1155/2008/186584 19132106 PMC2613969

[B99] Ohta T. Takahashi M. Ochiai A. (2006). Increased protein expression of both inducible nitric oxide synthase and cyclooxygenase-2 in human colon cancers. Cancer Lett. 239, 246–253 10.1016/j.canlet.2005.08.014 16216410

[B100] Oka K. Suzuki Y. Iida H. Nakano T. (2003). Pd-ECGF positivity correlates with better survival, while iNOS has no predictive value for cervical carcinomas treated with radiotherapy. Int. J. Radiat. Oncol. Biol. Phys. 57, 217–221 10.1016/S0360-3016(03)00436-X 12909236

[B101] Oronsky B. T. Knox S. J. Scicinski J. J. (2012). Is nitric oxide (NO) the last word in radiosensitization? a review. Transl. Oncol. 5, 66–71 22496921 10.1593/tlo.11307 PMC3323926

[B102] Ostrand-Rosenberg S. Sinha P. (2009). Myeloid-derived suppressor cells: linking inflammation and cancer. J. Immunol. 182, 4499–4506 10.4049/jimmunol.0802740 19342621 PMC2810498

[B103] Ou Yang K. X. Liang J. Huang Z. Q. (2011). Association of clinicopathologic parameters with the expression of inducible nitric oxide synthase and vascular endothelial growth factor in mucoepidermoid carcinoma. Oral. Dis. 17, 590–596 10.1111/j.1601-0825.2011.01813.x 21624013

[B104] Ozel E. Pestereli H. E. Simsek T. Erdogan G. Karaveli F. S. (2006). Expression of cyclooxygenase-2 and inducible nitric oxide synthase in ovarian surface epithelial carcinomas: is there any correlation with angiogenesis or clinicopathologic parameters? Int. J. Gynecol. Cancer 16, 549–555 10.1111/j.1525-1438.2006.00567.x 16681724

[B105] Pautz A. Art J. Hahn S. Nowag S. Voss C. Kleinert H. (2010). Regulation of the expression of inducible nitric oxide synthase. Nitric Oxide 23, 75–93 10.1016/j.niox.2010.04.007 20438856

[B106] Perske C. Lahat N. Levin S. S. Bitterman H. Hemmerlein B. Rahat M. A. (2010). Loss of inducible nitric oxide synthase expression in the mouse renal cell carcinoma cell line RENCA is mediated by microRNA miR-146a. Am. J. Pathol. 177, 2046–2054 10.2353/ajpath.2010.091111 20709800 PMC2947298

[B107] Pucci F. Venneri M. A. Biziato D. Nonis A. Moi D. Sica A. (2009). A distinguishing gene signature shared by tumor-infiltrating Tie2-expressing monocytes, blood “resident” monocytes, and embryonic macrophages suggests common functions and developmental relationships. Blood 114, 901–914 10.1182/blood-2009-01-200931 19383967

[B108] Puhakka A. Kinnula V. Napankangas U. Saily M. Koistinen P. Paakko P. (2003). High expression of nitric oxide synthases is a favorable prognostic sign in non-small cell lung carcinoma. APMIS 111, 1137–1146 10.1111/j.1600-0463.2003.apm1111210.x 14678024

[B109] Pukkila M. J. Kellokoski J. K. Virtaniemi J. A. Kumpulainen E. J. Johansson R. T. Halonen P. M. (2002). Inducible nitric oxide synthase expression in pharyngeal squamous cell carcinoma: relation to p53 expression, clinicopathological data, and survival. Laryngoscope 112, 1084–1088 10.1097/00005537-200206000-00027 12160278

[B110] Qian B. Z. Pollard J. W. (2010). Macrophage diversity enhances tumor progression and metastasis. Cell 141, 39–51 10.1016/j.cell.2010.03.014 20371344 PMC4994190

[B111] Rabinovich G. A. Gabrilovich D. Sotomayor E. M. (2007). Immunosuppressive strategies that are mediated by tumor cells. Annu. Rev. Immunol. 25, 267–296 10.1146/annurev.immunol.25.022106.141609 17134371 PMC2895922

[B112] Radi R. (2004). Nitric oxide, oxidants, and protein tyrosine nitration. Proc. Natl. Acad. Sci. U.S.A. 101, 4003–4008 10.1073/pnas.0307446101 15020765 PMC384685

[B113] Rahat M. A. Bitterman H. Lahat N. (2011). Molecular mechanisms regulating macrophage response to hypoxia. Front. Immunol. 2:45 10.3389/fimmu.2011.00045 22566835 PMC3342364

[B114] Rajnakova A. Moochhala S. Goh P. M. Ngoi S. (2001). Expression of nitric oxide synthase, cyclooxygenase, and p53 in different stages of human gastric cancer. Cancer Lett. 172, 177–185 10.1016/S0304-3835(01)00645-0 11566494

[B115] Ratovitski E. A. Alam M. R. Quick R. A. McMillan A. Bao C. Kozlovsky C. (1999a). Kalirin inhibition of inducible nitric-oxide synthase. J. Biol. Chem. 274, 993–999 9873042 10.1074/jbc.274.2.993

[B116] Ratovitski E. A. Bao C. Quick R. A. Mcmillan A. Kozlovsky C. Lowenstein C. J. (1999b). An inducible nitric-oxide synthase (NOS)-associated protein inhibits NOS dimerization and activity. J. Biol. Chem. 274, 30250–30257 10514518 10.1074/jbc.274.42.30250

[B117] Reiman J. M. Kmieciak M. Manjili M. H. Knutson K. L. (2007). Tumor immunoediting and immunosculpting pathways to cancer progression. Semin. Cancer Biol. 17, 275–287 10.1016/j.semcancer.2007.06.009 17662614 PMC2742305

[B118] Ridnour L. A. Isenberg J. S. Espey M. G. Thomas D. D. Roberts D. D. Wink D. A. (2005). Nitric oxide regulates angiogenesis through a functional switch involving thrombospondin-1. Proc. Natl. Acad. Sci. U.S.A. 102, 13147–13152 10.1073/pnas.0502979102 16141331 PMC1201580

[B119] Ridnour L. A. Thomas D. D. Donzelli S. Espey M. G. Roberts D. D. Wink D. A. (2006). The biphasic nature of nitric oxide responses in tumor biology. Antioxid. Redox Signal. 8, 1329–1337 10.1089/ars.2006.8.1329 16910780

[B120] Ridnour L. A. Windhausen A. N. Isenberg J. S. Yeung N. Thomas D. D. Vitek M. P. (2007). Nitric oxide regulates matrix metalloproteinase-9 activity by guanylyl-cyclase-dependent and -independent pathways. Proc. Natl. Acad. Sci. U.S.A. 104, 16898–16903 10.1073/pnas.0702761104 17942699 PMC2040425

[B121] Ropponen K. M. Kellokoski J. K. Lipponen P. K. Eskelinen M. J. Alanne L. Alhava E. M. (2000). Expression of inducible nitric oxide synthase in colorectal cancer and its association with prognosis. Scand. J. Gastroenterol. 35, 1204–1211 10.1080/003655200750056709 11145294

[B122] Sandes E. O. Faletti A. G. Riveros M. D. Vidal Mdel C. Gimenez L. Casabe A. R. (2005). Expression of inducible nitric oxide synthase in tumoral and non-tumoral epithelia from bladder cancer patients. Nitric Oxide 12, 39–45 10.1016/j.niox.2004.11.003 15631946

[B123] Schreiber R. D. Old L. J. Smyth M. J. (2011). Cancer immunoediting: integrating immunity's roles in cancer suppression and promotion. Science 331, 1565–1570 10.1126/science.1203486 21436444

[B124] Stout R. D. Suttles J. (2004). Functional plasticity of macrophages: reversible adaptation to changing microenvironments. J. Leukoc. Biol. 76, 509–513 10.1189/jlb.0504272 15218057 PMC1201486

[B125] Stout R. D. Watkins S. K. Suttles J. (2009). Functional plasticity of macrophages: *in situ* reprogramming of tumor-associated macrophages. J. Leukoc. Biol. 86, 1105–1109 10.1189/jlb.0209073 19605698 PMC2774875

[B126] Swana H. S. Smith S. D. Perrotta P. L. Saito N. Wheeler M. A. Weiss R. M. (1999). Inducible nitric oxide synthase with transitional cell carcinoma of the bladder. J. Urol. 161, 630–634 10.1016/S0022-5347(01)61985-2 9915473

[B127] Switzer C. H. Glynn S. A. Cheng R. Y. Ridnour L. A. Green J. E. Ambs S. (2012). S-Nitrosylation of EGFR and src activates an oncogenic signaling network in human basal-like breast cancer. Mol. Cancer Res. 10, 1203–1215 10.1158/1541-7786.MCR-12-0124 22878588 PMC3463231

[B128] Taylor B. S. De Vera M. E. Ganster R. W. Wang Q. Shapiro R. A. Morris S. M. Jr. (1998). Multiple NF-kappaB enhancer elements regulate cytokine induction of the human inducible nitric oxide synthase gene. J. Biol. Chem. 273, 15148–15156 10.1074/jbc.273.24.15148 9614127

[B129] Tschugguel W. Schneeberger C. Unfried G. Czerwenka K. Weninger W. Mildner M. (1999). Expression of inducible nitric oxide synthase in human breast cancer depends on tumor grade. Breast Cancer Res. Treat. 56, 145–151 10.1023/A:1006288526311 10573107

[B130] Vakkala M. Kahlos K. Lakari E. Paakko P. Kinnula V. Soini Y. (2000). Inducible nitric oxide synthase expression, apoptosis, and angiogenesis in *in situ* and invasive breast carcinomas. Clin. Cancer Res. 6, 2408–2416 10873093

[B131] Venneri M. A. De Palma M. Ponzoni M. Pucci F. Scielzo C. Zonari E. (2007). Identification of proangiogenic TIE2-expressing monocytes (TEMs) in human peripheral blood and cancer. Blood 109, 5276–5285 10.1182/blood-2006-10-053504 17327411

[B132] Vesely M. D. Kershaw M. H. Schreiber R. D. Smyth M. J. (2011). Natural innate and adaptive immunity to cancer. Annu. Rev. Immunol. 29, 235–271 10.1146/annurev-immunol-031210-101324 21219185

[B133] Vickers S. M. Macmillan-Crow L. A. Green M. Ellis C. Thompson J. A. (1999). Association of increased immunostaining for inducible nitric oxide synthase and nitrotyrosine with fibroblast growth factor transformation in pancreatic cancer. Arch. Surg. 134, 245–251 10.1001/archsurg.134.3.245 10088562

[B134] Walmsley S. R. Chilvers E. R. Whyte M. K. (2009). Hypoxia. Hypoxia, hypoxia inducible factor and myeloid cell function. Arthritis Res. Ther. 11, 219 10.1186/ar2632 19435530 PMC2688173

[B135] Wang B. Wei D. Crum V. E. Richardson E. L. Xiong H. H. Luo Y. (2003). A novel model system for studying the double-edged roles of nitric oxide production in pancreatic cancer growth and metastasis. Oncogene 22, 1771–1782 10.1038/sj.onc.1206386 12660813

[B136] Wang X. Zhao Q. Matta R. Meng X. Liu X. Liu C. G. (2009). Inducible nitric-oxide synthase expression is regulated by mitogen-activated protein kinase phosphatase-1. J. Biol. Chem. 284, 27123–27134 10.1074/jbc.M109.051235 19651781 PMC2785641

[B137] Weigert A. Brune B. (2008). Nitric oxide, apoptosis and macrophage polarization during tumor progression. Nitric Oxide 19, 95–102 10.1016/j.niox.2008.04.021 18486631

[B138] Wink D. A. Hines H. B. Cheng R. Y. Switzer C. H. Flores-Santana W. Vitek M. P. (2011). Nitric oxide and redox mechanisms in the immune response. J. Leukoc. Biol. 89, 873–891 10.1189/jlb.1010550 21233414 PMC3100761

[B139] Wyckoff J. Wang W. Lin E. Y. Wang Y. Pixley F. Stanley E. R. (2004). A paracrine loop between tumor cells and macrophages is required for tumor cell migration in mammary tumors. Cancer Res. 64, 7022–7029 10.1158/0008-5472.CAN-04-1449 15466195

[B140] Xie Q. Nathan C. (1994). The high-output nitric oxide pathway: role and regulation. J. Leukoc. Biol. 56, 576–582 7525816 10.1002/jlb.56.5.576

[B141] Yang L. Guo A. Gu J. C. (2011). c-Jun N-terminal kinase and nuclear factor kappaB mediate nitric oxide-induced expression of matrix metalloproteinase-13. Int. Orthop. 35, 1261–1266 10.1007/s00264-010-1056-y 20524115 PMC3167433

[B142] Yasuda H. (2008). Solid tumor physiology and hypoxia-induced chemo/radio-resistance: novel strategy for cancer therapy: nitric oxide donor as a therapeutic enhancer. Nitric Oxide 19, 205–216 10.1016/j.niox.2008.04.026 18503779

[B143] Ying L. Hofseth A. B. Browning D. D. Nagarkatti M. Nagarkatti P. S. Hofseth L. J. (2007). Nitric oxide inactivates the retinoblastoma pathway in chronic inflammation. Cancer Res. 67, 9286–9293 10.1158/0008-5472.CAN-07-2238 17909036 PMC2752153

[B144] Zafirellis K. Zachaki A. Agrogiannis G. Gravani K. (2010). Inducible nitric oxide synthase expression and its prognostic significance in colorectal cancer. APMIS 118, 115–124 10.1111/j.1600-0463.2009.02569.x 20132175

[B145] Zhang J. Peng B. Chen X. (2005). Expressions of nuclear factor kappaB, inducible nitric oxide synthase, and vascular endothelial growth factor in adenoid cystic carcinoma of salivary glands: correlations with the angiogenesis and clinical outcome. Clin. Cancer Res. 11, 7334–7343 10.1158/1078-0432.CCR-05-0241 16243805

[B146] Zhang W. He X. J. Ma Y. Y. Wang H. J. Xia Y. J. Zhao Z. S. (2011). Inducible nitric oxide synthase expression correlates with angiogenesis, lymphangiogenesis, and poor prognosis in gastric cancer patients. Hum. Pathol. 42, 1275–1282 10.1016/j.humpath.2010.09.020 21333324

[B147] Ziche M. Morbidelli L. (2009). Molecular regulation of tumour angiogenesis by nitric oxide. Eur. Cytokine Netw. 20, 164–170 10.1684/ecn.2009.0169 20167555

